# Assessment of sanitary conditions of unregistered pig slaughter slabs and post mortem examination of pigs for *Taenia solium* metacestodes in Kaduna metropolis, Nigeria

**DOI:** 10.1186/2049-9957-3-45

**Published:** 2014-12-03

**Authors:** Agnes U Edia-Asuke, Helen I Inabo, Veronica J Umoh, Clement MZ Whong, Sunday Asuke, Richard E Edeh

**Affiliations:** Department of Microbiology, Ahmadu Bello University, Zaria, Kaduna Nigeria; Biological Sciences, University of KwaZulu-Natal, Westville Campus, Durban, South Africa; Department of Community Medicine, Ahmadu Bello University Teaching Hospital, Shika Zaria, Kaduna Nigeria; Faculty of Veterinary Medicine, Ahmadu Bello University, Zaria, Kaduna Nigeria

**Keywords:** Pig slaughter slabs, Home slaughter, Copro-Ag ELISA, *Taenia solium* metacestodes, Kaduna, Nigeria

## Abstract

**Background:**

A number of studies document the prevalence of *Taenia solium* infections in Nigeria, yet these studies do not cover porcine cysticercosis in private home slaughter slabs where there is no routine meat inspection and backyard pig keeping, slaughtering and sale are common practice.

**Methods:**

An environmental and sanitary assessment was conducted within two unregistered home pig slaughter slabs in selected parts of the Kaduna metropolis in Nigeria. Slaughter premises were inspected for availability of basic facilities and questionnaires were used to elicit necessary informative data. Butchers were examined for taeniasis by stool microscopy and copro-antigen enzyme linked immunosorbent assay (copro-Ag ELISA) to ascertain *T. solium* – taeniasis. Pigs slaughtered at the premises were examined for cysticerci.

**Results:**

Home slaughter conditions were substandard, unhygienic and lacked the basic facilities of a proper slaughterhouse. Prevalence of porcine cysticercosis was 9.3%. The butchers participating in the study had very poor knowledge of *T. solium* infections and 30% tested positive for taeniasis by copro-Ag ELISA at the time of the study.

**Conclusion:**

Home slaughter of pigs in the areas studied should be considered and integrated as a component of prevention and control programmes – particularly through educational interventions – in order to equip individuals involved with a good understanding of the risks associated with animal husbandry and human practices.

**Electronic supplementary material:**

The online version of this article (doi:10.1186/2049-9957-3-45) contains supplementary material, which is available to authorized users.

## Multilingual abstracts

Please see Additional file 1 for translations of the abstract into the six official working languages of the United Nations.

## Background

*Taenia solium* is an important parasite that causes serious public health and socio-economic challenges, particularly in endemic regions where pork is often consumed and hygiene standards are low. The parasite has the ability to infect both humans (causing neurocysticercosis [NCC] and taeniasis) and pigs (causing porcine cysticercosis). Both humans and pigs can develop cysticercosis if they ingest *T. solium* ova passed directly through human stool or by a faecal oral route involving consumption of food or water contaminated with ova from faecal matter. The larval stage or metacestode of *T. solium* is responsible for cysticercosis in both humans and pigs. The metacestodes form fluid-filled sacs called cysts or cysticerci. *T. solium* cysticerci lodge in a number of internal organs and musculature of its hosts, however, in humans, when the cysticerci are lodged in the central nervous system (CNS), NCC results. Reported to be the most important parasitic disease affecting the nervous system, NCC accounts for about 30% of all acquired epilepsy cases in endemic areas [1], indicating strong public health significance. Cysticercosis and taeniasis have not been extensively studied and understood in Nigeria, however, it has been reported that extensive data is available in other African countries such as Benin, Togo, Cameroon, Tanzania, South Africa and Zambia [2–5]. Nigeria has an existing abattoir law that is meant to ensure proper management and livestock slaughtering to protect people’s health. Despite this, production, handling, sales and consumption of poor quality animal food products have been identified as a serious public health problem, which results from a confluence of several factors affecting different segments of the livestock industry [6]. The law forbids the slaughter of livestock for human consumption in locations other than abattoirs, however, home slaughter and sale of pork is a common practice in parts of the Kaduna metropolis. The exact proportion of registered pig slaughter slabs in Kaduna is not known; a field survey records 46 animal slaughter slabs including pigs [7], however, based on observation, unregistered home slaughter slabs seem to be on the rise. The lack of regular meat inspection, particularly in unregistered slaughter premises, has the tendency to promote the sale and consumption of unwholesome pork products that pose a health risk to consumers. It is reported that at such illegal premises in Cameroon, infected pork is usually sold at a decreased price [2]. There is a need for a better understanding of issues surrounding pork production, handling, consumption and quality assessment/meat inspection in homes, and how these factors impact on the transmission of *T. solium*, thereby exacerbating the cysticercosis-taeniasis complex. This survey was therefore designed to assess sanitary conditions of two selected home pig slaughter slabs, and consequently determine poor hygiene practices and knowledge of butchers working at, as well as detect porcine cysticercosis in pigs slaughtered and sold within, these selected sites.

## Methods

### Study area

This study was conducted in the Nasarawa and Gonin Gora communities located within the Chikun Local Government Area of the Kaduna metropolis, Nigeria. The Kaduna metropolis has an overall population of 1,570,331, with a household population of 314, 066 [8, 9]. Both communities are involved in subsistence farming and rearing of livestock such as cattle, sheep, goats and pigs for both consumption and income generation. The choice of the communities was based on their involvement in small backyard pig farming and pork consumption as observed during a pilot survey conducted within these communities.

### Ethical permit

This research was conducted in compliance with the Helsinki declaration. The ethical clearance was granted by The Kaduna State Ministry of Health (MOH/ADM/744/VOL.1/).

### Study design

This was a qualitative study, whereby data was generated with the aid of self-administered questionnaires and field observations. A structured questionnaire was administered to the butchers responsible for slaughtering and processing the raw pork for consumption and sale. The questionnaire was designed to provide necessary information about slaughter slab conditions, management and available facilities, as well as the socio-demographic factors, knowledge and behaviour of the butchers with respect to the public health significance of *T. solium*. Nasarawa and Gonin Gora were selected by simple random sampling, however, the selection of the exact slaughter slabs was based on willingness of the respondents to participate in the study.

### Sanitary assessment of the pig slaughter slabs

The selected slaughter slabs were visited once every week for a period of six months between January and July 2013 at slaughtering times for post-mortem inspection of the pig carcasses and environmental and sanitary inspections. The visits were scheduled to coincide with the slaughter timetables provided by the butchers.

### Environmental inspection and examination of human faecal samples for *Taenia solium*

The slaughter premises were inspected to elicit the following information: the presence of necessary facilities such as structure/type of pig abattoir, location of the slaughter slab, average number of pigs slaughtered per day, method of slaughtering, pig sources and availability of veterinary services, water source, the availability of a toilet/drainage facility and whether routine meat inspection was conducted. Environmental samples comprising 25 water samples, 50 soil samples (25 soil samples from each of the premises) and 15 open drainage samples were collected from within and around the slaughter premises and examined for taeniid ova following standard microscopic procedures [10, 11]. Butchers handling pork at the slaughter sites were interrogated and observed in order to ascertain their knowledge, attitudes and behavioural practices in relation to their awareness of the mechanisms of transmission, prevention and public health significance of cysticercosis and taeniasis. The faecal samples of ten butchers present at both slaughter premises were examined for *T. solium* by both coprology using the formalin ether technique [10], and the copro-Ag ELISA technique using polyclonal antibodies to detect the presence of copro antigens [6, 12].

### Meat inspection

Post-mortem meat inspection of 43 pig carcasses was conducted by visual inspection/examination of the carcasses, and incisions and inspection of cyst predilection sites, which comprised tongues, masseter and thigh muscles, livers, lungs and heart. Neck and shoulder muscles, which comprised non-routine predilection sites, were also examined [13]. Live cysts were identified as fluid-filled, translucent with invaginated visible scolices [13, 14], and then counted.

## Results

### Description of available facilities and sanitary conditions at slaughter premises

The two slaughter premises were basically part of the yards of the properties where the owners resided. They appeared to be principally low-grade, and lacking in standard slaughter facilities and basic amenities such as proper drainage channels and pipe-borne water. Wells served as the main water source at both slaughter premises. Slaughtering and dressing of carcasses was done under unsanitary conditions on the bare concrete floors. Both slaughter sites practiced free ranging/backyard pig keeping without routine veterinary and meat inspection; the Nasarawa slab also sourced pigs from semi-intensive farms (Table 1 shows the available facilities and management structures at the slaughter premises). Environmental assessment revealed that water stored for domestic purposes in the two slaughter premises was not contaminated with taeniid ova. One out of the 25 soil samples strategically collected from the environs of the Nasarawa slaughter slab was positive with taeniid ova by microscopic examination, while the 25 soil samples from the Gonin Gora premises and all drainage samples examined tested negative.

**Table 1:** **Facilities, sanitary conditions and management of two unregistered pig slaughter slabs in the Kaduna metropolis, Nigeria**

*Facilities and management*	*Slaughter slab 1*	*Slaughter slab 2*
*Toilet system available at premises*	Pit latrine	Pit latrine
*Structure of slaughter premises*	Makeshift, bare concrete floor inside the residential yard of the owner	Makeshift, cemented floor used as the slab at the backyard of the house, opposite a refuse dump site
*Water source at premises*	Well	Well
*Drainage facility/waste disposal*	Improvised open drainage	Open drainage
*Frequency of pig slaughtering*	Once a week	Once a week
*Number of pigs slaughtered per day*	5–6 pigs per day	1 pig per day
*Total number of pigs slaughtered during the survey period*	32	11
*Pig source/management system at point source*	Semi-intensive farms and free ranging pigs	Free ranging pigs
*Availability of vet services at source*	Routinely at intensive farms, seldom for free range pigs	Seldom
*Routine meat inspection at slaughter slabs by official authorities*	Absent	Absent
*Use of sanitizers for cleaning*	No	No

Key: Slaughter slab 1: located at Nasarawa.

Slaughter slab 2: located at Gonin Gora.

### Socio-demographics, behaviour and medical history of the butchers

Questionnaire data revealed that all participating butchers were males and only 20% had a formal education. The majority (80%) of the butchers had poor knowledge of *T. solium* infections and its public health significance. Hygiene was poor among the butchers: they did not treat their drinking water, did not wear hand gloves or aprons on duty and 70% did not wash hands regularly after toilet use. None of the butchers had any history of epilepsy nor did any of them deworm themselves regularly, however, copro-Ag ELISA revealed that 30% had taeniasis at the time of the study (see Tables 2 and 3 for the socio-demographics, behaviour and medical histories of the butchers).

**Table 2:** **Socio-demographic factors and medical history of butchers (in relation to cysticercosis) at selected slaughter slabs**

*Socio-demographic factors and medical history*		*Outcome n = 10*
*Sex:*	M	10 (100%)
	F	0
*Marital status:*	Married	5 (50%)
	Single	5 (50%)
*Educational level:*	Informal	8 (80%)
	Formal	2 (20%)
*History of epilepsy:*	Yes	0
	No	10 (100%)
*Regular deworming of self*:	Yes	0
	No	10 (100%)
*Tested positive to taeniasis by copro-Ag ELISA at time of study*:		
	Yes	3 (30%)*
	No	7 (70%)

Key: copro-Ag ELISA: Copro-antigen detection by enzyme linked immunosorbent assay.

*Only one tested positive by coproscopic examination.

**Table 3:** **Knowledge, behavioural and hygiene practices of butchers in relation to cysticercosis at selected slaughter slabs**

*Factors*		*Outcome (n = 10)*
*Boiling/treatment of drinking water:*	Yes	0
	No	10 (100%)
*Regular hand washing after toilet use*:	Yes	3 (30%)
	No	7 (70%)
*Regular hand washing after handling pork with:*	Soap	9 (90%)
	Sanitizer	0
	Water only	1 (10%)
*Wearing of hand gloves and aprons at work:*	Yes	0
	No	10 (100%)
*Consumption of pork:*	Yes	10 (100%)
	No	0
*Roasting and eating pork while handling raw pork:*	Yes	4 (40%)
	No	6 (60%)
*Method of pork processing for consumption:*	Cooked	4 (40%)
	Cooked and fried	2 (20%)
	Roasted	4 (40%)
*Knowledge on public health significance of Taenia solium:*	Yes	0
	No	10 (100%)

### Post-mortem examination of pig carcasses at slaughter slabs

Out of the total 43 pigs examined by post-mortem inspection at the two unregistered slaughter slabs, four were positive for viable cysticerci, giving an overall prevalence of 9.3%. The pigs examined at Nasarawa and Gonin Gora slaughter slabs had a cysticercosis prevalence of 9.4% (n = 32) and 9.1% (n = 11), respectively. A total of 34 cysticerci were counted from common predilection sites, which included tongue, masseter, thigh muscles and the heart, and also from the neck and shoulder muscles which are uncommon predilection sites. No cysticerci were recovered from the lungs or liver of all carcasses examined. The highest number of cysticerci was recovered from the neck and shoulder muscles 11 (32.4%), followed by the heart 9 (26.5%), thigh muscles 7 (20.6%), tongue 4 (11.8%) and masseter muscles 3 (8.8%), respectively.

## Discussion

It is reported that – under normal circumstances – many abattoirs and slaughter slabs in developing countries are poorly constructed, and have poor slaughter and meat inspection facilities [15]. As in other studies done in Nepal [16], Tanzania [17] and Imo State, Nigeria [18], this study reported poor hygienic and substandard conditions at the two home slaughter slabs. Home slaughter conditions are substandard because they are makeshift and not constructed to actually meet the requirements of ideal slaughter premises. Such substandard conditions lead to ease of microbial contamination because proper slaughter facilities are lacking and thus hygiene is poor. For instance, poor sewage and open drainages observed in the study led to the brooding of flies which could contaminate food and water within the premises. The lack of pipe-borne water in this study supports similar findings in Zuru, Nigeria and Nepal, where wells served as major sources of water in slaughter slabs [11, 16]; water from such alternative sources could be contaminated on account of direct exposure to environmental factors. The free roaming of pigs and lack of routine veterinary services observed in this study are of great concern; inadequate care of food animals reduces their productivity and exposes them to disease agents, which may become hazardous to the animals, and to humans and their environment [18, 19]. The health and proper husbandry of livestock is therefore essential for optimisation of livestock productivity, as well as for promoting good health in human consumers [18]. Lack of meat inspection is critical in the transmission of *T. solium* to humans [11] and exacerbates the cysticercosis-taeniasis complex. Usually, routine meat inspection is lacking in home slaughtering, and should be considered an important predisposing factor because of the risk it poses for creating an urban foci or point source of infection, as the case may be in this study.

The low recovery of taeniid ova from environmental samples in this survey does not necessarily reflect the true status of the density of taeniid ova in the environment. Harsh environmental conditions could distort identifiable morphological features of the ova, and, consequently, the under reporting of positive cases in the environmental samples examined microscopically [11].

In Nigeria, it is believed that males, illiterates and school dropouts dominate butchery [18]. This study agrees with studies conducted in Nepal and Ghana, where it was reported that 80% and 64% of butchers, respectively, had no formal education [16, 20]. Knowledge plays a key role in the prevention and control of diseases. Health education intervention studies in Mexico and Southern Tanzania revealed that knowledge of *T. solium* played a significant role in preventing transmission in the human-pig cycle [21, 22]. The poor knowledge of *T. solium* among the butchers in this study was evident in their behaviour, which included poor hand washing practices, not treating drinking water and handling of raw pork with bare hands without the use of hand gloves or aprons. A similar finding in Nepal also reported that butchers neither wore gloves nor masks while handling raw meat and only 30% wore an apron regularly at work [16]. In addition to knowledge, not using protective covering could also be attributed to the discomfort it brings. Soaps, sanitizers and detergents are known to clean surfaces thoroughly and also reduce microbial population on contaminated hands, however, they weren’t being used by the butchers in this study. Butchers with taeniasis in this study pose a serious risk of cysticercosis both to themselves and to others in close contact with them. Household members should be aware of this in order to prevent further spread of infection, especially as hand washing after using the toilet wasn’t common practice among the butchers. Despite the prevalence of taeniasis reported among the butchers in this study, there was no reported history of epilepsy and this relates to the fact that having taeniasis is not always indicative of the presence of cysticercosis-related epilepsy. However, it has been reported that the most important risk factor for acquiring cysticercosis is the proximity of a tapeworm carrier [23], hence individuals with cysticercosis stand a higher risk of developing epilepsy.

The reported 9.3% prevalence in this study is higher than the 2.5% reported in Nsukka, Nigeria [14], closer to the 9.5% reported in Northern Nigeria [24] and much lower than 14.40% reported in Zuru [11]. Higher prevalence rates have been reported in Cameroon and South Africa [2, 4]. These varying prevalence rates are attributed to the diagnostic techniques used. The tongue examination technique in live pigs is said to have a lower sensitivity than the post-mortem meat inspection [11], although this is said to be 100% specific [25]. This is because several predilection sites are examined at post mortem and incisions are made where necessary, in contrast to only in the tongue in lingual palpation [11]. Detailed necroscopy has been recommended as a more reliable diagnostic method in pigs with low infection [25], but this is impractical for routine inspection because it is time consuming, expensive and leads to commercial losses [26, 27]. The absence of cysticerci in the liver and lungs of pigs in this study concurs with the findings of a similar study done in Nsukka [14], but contradicts the findings of a survey done in Zuru [11], where cysts were found in the liver and lungs during routine meat inspection. The findings of this study support the high cystic affinity for neck and shoulder muscles, which are non-routine predilection sites, and for muscles of the heart, tongue and masseter reported in other studies as common predilection sites [25, 28].

## Conclusion

The two selected home slaughter slabs in Nasarawa and Gonin Gora were substandard, operating under unhygienic conditions, lacking in basic facilities and, most importantly, lacking in routine meat inspection. Given the 9.3% prevalence of cysticercoid pigs, and the poor knowledge of *T. solium* infections, poor hygiene and 30% taeniasis prevalence among the butchers operating these home slaughter premises, there is a high risk of exposure to taeniasis and cysticercosis in these homes. Home slaughter should therefore be considered a potential component of intervention and control programmes in order to equip individuals involved with a better understanding of the risks associated with animal husbandry and human behavioural practices.

## Electronic supplementary material

Additional file 1: **Multilingual abstracts in the six official working languages of the United Nations.** (PDF 343 KB)

## Abbreviations

Copro-Ag ELISA: Copro-antigen enzyme linked immunosorbent assay
CNS: Central nervous system
NCC: Neurocysticercosis
NPC: National Population Commission
FRN: Federal Republic of Nigeria.
