# Supplementary material for: Assessment of sanitary conditions of unregistered pig slaughter slabs and post mortem examination of pigs for Taenia solium metacestodes in Kaduna metropolis, Nigeria
Source: Infect Dis Poverty. 2014 Dec 3;3:45. doi: 10.1186/2049-9957-3-45 (PMC4322547; doi:10.1186/2049-9957-3-45)
Supplement: Supplementary file 1 — Additional file 1: Multilingual abstracts in the six official working languages of the United Nations. (PDF 343 KB) [file 40249_2014_83_MOESM1_ESM.pdf]

Translation of the abstract into the six official working languages of the United Nations

تقييم الظروف الصحية لذبح الخنازير خارج المجازر المرخصة وفحص الخنازير بعد الوفاة للتأكد من إصابتها بالأطوار اليرقية للديدان الشريطية ***Taenia solium*** في مدينة كادونا، نيجيريا

إيديا-أسوكي أ و، إينابو ه ي، أوموه ف ج، وهونج س م ز، أسوكي س، إيديه إ ر

المستخلص

خلفية: توثق عدد من الدراسات انتشار عدوى *Taenia solium* في نيجيريا، ولكن هذه الدراسات لا تغطي داء الكيسات المذنبة في الخنازير التي تذبح في المجازر المنزلية حيث لا يتم فحص لحوم الخنزير بصورة روتينية وحيث يشيع تربية وذبح وبيع لحم الخنزير في مثل هذه المجازر غير المرخصة.

الطرق: تم إجراء تقييم بيئي وصحي على مدار سنتين لمجزرين منزليين غير مرخصين في منطقتين مختارتين من مدينة كادونا في نيجيريا. تم تفتيش أماكن الذبح للتحقق من مدى توافر المرافق الأساسية بها واستخدمت الاستبيانات للحصول على المعلومات اللازمة. تم عمل تحاليل للجزارين للتحقق من إصابتهم بالشريطيات بواسطة فحص البراز بالمجهر واختبار copro-Ag ELISA للتأكد من عدم إصابتهم بـ *T. solium*- داء الشريطيات. تم فحص الخنازير المذبوحة للتحقق من عدم إصابتها باللاسلكيات المذنبة.

النتائج: كانت ظروف الذبح في تلك المجازر دون المستوى المطلوب، حيث كانت غير صحية وتفتقر إلى المرافق الأساسية للمجزر السليم. كانت نسبة انتشار داء الكيسات المذنبة الخنازير 9,3%. وكانت درجة وعي الجزارين المشاركين في الدراسة بعدوى *T. solium* محدودة للغاية وتبين وجود نسبة 30٪ إيجابية ومصابة بالشريطيات بواسطة اختبار copro-Ag ELISA في وقت الدراسة.

الاستنتاج: ينبغي النظر في ذبح الخنازير في المجازر المنزلية غير المرخصة في المناطق المدروسة وإدماجها ضمن برامج الوقاية والمكافحة - لا سيما من خلال التدخلات التعليمية - من أجل تعريف الأفراد المعنيين بالمخاطر المتعلقة بتربية الحيوانات والممارسات البشرية المرتبطة بها.

Translated from English version into Arabic by Saher Salama, through

TRANSLATORS
WITHOUT BORDERS

尼日利亚卡杜纳市未注册生猪屠宰场卫生条件评估和活猪经宰杀尸检其中绦期猪带绦虫感染情况研究

Edia-Asuke AU, Inabo HI, Umoh VJ, Whong CMZ, Asuke S, Edeh ER

摘要

引言：许多研究证明猪带绦虫病在尼日利亚感染流行，但这些研究并未涵盖私人家庭式屠宰场所中的猪囊尾蚴病，在这些屠宰场内没有常规肉类检验，在其后院饲养、屠宰并销售活猪是很常见的做法。

方法：在尼日利亚卡杜纳市的选定区域中两个未注册的家庭生猪屠宰场进行环保和卫生评估。检查屠宰场的基础设施，并通过问卷调查获取必要信息数据。对屠夫进行粪检和粪抗原的酶联免疫吸附测试（ELISA 法）。活猪经宰杀尸检其猪囊尾蚴感染情况。

结果：家庭屠宰场的卫生条件不合格，不卫生且缺乏配套的基本设施。猪囊尾蚴病的患病率为 9.3%。参与研究的屠夫对猪带绦虫病相关知识所知甚少，研究期间 30%屠夫的猪带绦虫病 ELISA 检测呈阳性。

结论：在所研究地区的家庭生猪屠宰场应考虑并整合为预防和控制方案的一部分 ，尤其是通过教育干预，使这些个人能够很好理解在畜牧业和其屠宰行为中的相关风险。

Translated from English version into Chinese by Zheng Qi, through

CHINESE CENTER FOR DISEASE CONTROL AND PREVENTION
NATIONAL INSTITUTE OF PARASITIC DISEASES

Evaluation des conditions sanitaires sur des lieux d'abattage à domicile non enregistrés et examen post-mortem des porcs pour recherche de *Taenia solium* metacestodes (ver solitaire) dans la métropole de Kaduna, au Nigeria.

Edia-Asuke AU, Inabo HI, Umoh VJ, Whong CMZ, Asuke S, Edeh ER

Résumé

Contexte: Des études indiquent la prévalence d'infections par le ver solitaire (*Tænia solium)* au Nigeria, cependant ces études n'abordent pas la cysticercose porcine dans les lieux d'abattage à domicile privés, dans lesquels il n'y a pas d'inspection de routine de la viande, et où élever des porcs dans la cour, puis les abattre et les vendre, est une pratique courante.
Méthodes: une évaluation sanitaire et environnementale a été faite sur deux lieux non enregistrés d'abattage à domicile de porcs dans certaines parties de la métropole de Kaduna au Nigeria. Les locaux d'abattage ont été inspectés afin de voir s'ils disposaient des installations élémentaires, et des questionnaires ont été soumis pour obtenir les données informatives nécessaires. Les bouchers ont eu une analyse microscopique des selles et un essai d'immuno-absorption enzymatique (copro-Ag ELISA) pour vérifier la présence de *T. solium* – taeniasis. Les porcs abattus dans les locaux ont été examinés pour une recherche de cysticercose.
Résultats: les conditions d'abattage à domicile ne correspondaient pas aux normes en vigueur, étaient insalubres et ne disposaient pas des installations élémentaires d'un abattoir correct. La prévalence de cysticercose porcine était de 9%. Les bouchers participant à cette étude avaient de très faibles notions des infections par le T. solium et 30% se sont révélés positifs au taeniasis par copro-Ag ELISA au cours de l'étude.
Conclusion: l'abattage à domicile de porcs dans les régions étudiées devraient être considéré et intégré comme composante des programmes de prévention et de contrôle – particulièrement par des interventions éducatives – afin de fournir aux personnes impliquées une bonne compréhension des risques associés à l'élevage animal et aux pratiques humaines.

Translated from English version into French by Ode Laforge, through

TRANSLATORS
WITHOUT BORDERS

Оценка санитарного состояния незарегистрированных свинобоен и исследование свиней после забоя на наличие плероцеркоидов свиного цепня в г. Кадун, Нигерия

А. У. Эдиа-Асуке, Х. И. Инабо, В. Д. Умо, С. М. З. Вонг, С. Асуке, Е. Р. Иде

Краткий обзор

История вопроса: Большое количество исследований подтверждают широкое распространение инфицирования свиным цепнем в Нигерии, в то же время эти исследования не рассматривают цистицеркоз свиней в частных домашних свинобойнях, где не проводится регулярная инспекция мяса, а содержание и забой свиней на заднем дворе является общепринятой практикой.

Методы: Была проведена экологическая и санитарная оценка в двух незарегистрированных домашних свинобойнях, которые находятся на территории г. Кадун, Нигерия. Была проведена инспекция помещений, в которых производится забой свиней, на наличие элементарного оборудования. Для получения необходимой информации использовались опросные листы. Мясников обследовали на тениоз при помощи микроскопического исследования кала и твердофазного иммуноферментного анализа на копро-антиген, чтобы выявить присутствие свиного цепня. Свиней, забой которых производился в обследуемых помещениях, проверили на наличие цистицерка.

Результаты: Домашние условия, в которых производился забой, не соответствовали стандартам, не имели элементарного оборудования, и их санитарное состояние было очень низким. Показатель инфицирования цистицеркозом составил 9,3%. Мясники, принявшие участие в исследовании, имели слабое представление о свином цепне. Проведенный иммуноферментный анализ на копро-антиген показал, что 30% обследованных были заражены тениозом.

Заключение: Домашний забой свиней в исследуемых районах необходимо рассмотреть для включения в программы профилактики и контроля, в частности, образовательные программы для того, чтобы информировать людей, занятых в этом секторе, о рисках, связанных с занятостью людей в животноводстве.

Translated from English version into Russian by Natalia.A, through

TRANSLATORS
WITHOUT BORDERS

Evaluación de las condiciones sanitarias de las losas no registradas para la matanza del cerdo y análisis post-mortem de éstos para la detección de *metacéstodos de Taenia solium* en la ciudad de Kaduna, Nigeria

Edia-Asuke AU , Inabo HI , Umoh, VJ , Whong CMZ , Asuke, Edeh ER

Resumen

Antecedentes: Un número de estudios documentan la prevalencia de infección por Taenia solium en Nigeria, sin embargo, estos estudios no cubren la cisticercosis porcina en las losas de matanzas en casas donde no hay inspecciones de carne rutinarias y la cría, matanza y venta cerdos constituyen una práctica común.

Métodos: Se realizó una evaluación ambiental y sanitaria en losas no registradas de matanza de cerdo de dos hogares de determinadas partes de la ciudad de Kaduna en Nigeria. Se inspeccionaron los locales de matanza para determinar la disponibilidad de servicios básicos y se utilizaron cuestionarios para obtener los datos necesarios de carácter informativo. Se examinó a los carniceros de teniasis mediante un microscopio para excrementos y se realizó un Ensayo por Inmunoabsorción Ligado a Enzimas (copro-Ag ELISA) para verificar la existencia de *T. solium* - teniasis. Se realizaron análisis de cisticerco a los cerdos sacrificados en los locales.

Resultados : Las condiciones de los mataderos resultaron ser precarias, insalubres y carecían de los servicios básicos propios de un buen matadero. Prevalencia de cisticercosis porcina del 9,3 %. Los carniceros participantes en el estudio tenían muy poco conocimiento de las infecciones por *T. solium* y el 30% dio positivo en teniasis por copro-Ag ELISA en el momento del estudio.
Conclusión: Los mataderos de cerdos en las zonas estudiadas deben ser considerados e integrados como un componente de los programas de prevención y control, en particular mediante intervenciones educativas, con el fin de proporcionar un buen entendimiento de los riesgos asociados con la ganadería y las prácticas humanas.

Translated from English version into Spanish by Sonsoles Marín, through

TRANSLATORS
WITHOUT BORDERS
